# Combination Approaches to Target PD-1 Signaling in Cancer

**DOI:** 10.3389/fimmu.2022.927265

**Published:** 2022-07-14

**Authors:** Emily K. Moore, Marianne Strazza, Adam Mor

**Affiliations:** ^1^ Division of Rheumatology, Department of Medicine, Columbia University Medical Center, New York, NY, United States; ^2^ Columbia Center for Translational Immunology, Columbia University Medical Center, New York, NY, United States; ^3^ Herbert Irving Comprehensive Cancer Center, Columbia University Medical Center, New York, NY, United States

**Keywords:** T cell, PD-1, SHP2, ITK, PD-L1

## Abstract

Cancer remains the second leading cause of death in the US, accounting for 25% of all deaths nationwide. Immunotherapy techniques bolster the immune cells’ ability to target malignant cancer cells and have brought immense improvements in the field of cancer treatments. One important inhibitory protein in T cells, programmed cell death protein 1 (PD-1), has become an invaluable target for cancer immunotherapy. While anti-PD-1 antibody therapy is extremely successful in some patients, in others it fails or even causes further complications, including cancer hyper-progression and immune-related adverse events. Along with countless translational studies of the PD-1 signaling pathway, there are currently close to 5,000 clinical trials for antibodies against PD-1 and its ligand, PD-L1, around 80% of which investigate combinations with other therapies. Nevertheless, more work is needed to better understand the PD-1 signaling pathway and to facilitate new and improved evidence-based combination strategies. In this work, we consolidate recent discoveries of PD-1 signaling mediators and their therapeutic potential in combination with anti-PD-1/PD-L1 agents. We focus on the phosphatases SHP2 and PTPN2; the kinases ITK, VRK2, GSK-3, and CDK4/6; and the signaling adaptor protein PAG. We discuss their biology both in cancer cells and T cells, with a focus on their role in relation to PD-1 to determine their potential in therapeutic combinations. The literature discussed here was obtained from a search of the published literature and ClinicalTrials.gov with the following key terms: checkpoint inhibition, cancer immunotherapy, PD-1, PD-L1, SHP2, PTPN2, ITK, VRK2, CDK4/6, GSK-3, and PAG. Together, we find that all of these proteins are logical and promising targets for combination therapy, and that with a deeper mechanistic understanding they have potential to improve the response rate and decrease adverse events when thoughtfully used in combination with checkpoint inhibitors.

## Introduction

Cancer immunotherapies represent an emergent yet powerful therapeutic paradigm, due to both their durable clinical responses and their applicability to a wide variety of tumors. Immune checkpoint therapies block inhibitory receptors on T cells, augmenting anti-tumor immune responses. Programmed cell death protein 1 (PD-1) is a critical inhibitory checkpoint for T cells, and antibodies that block ligand binding free the T cells to identify and clear malignant tumor cells. As such, PD-1 is the subject of significant testing, with 786 completed and 4,897 ongoing clinical trials targeting it (1). Despite the striking success of these antibodies, most patients do not respond to PD-1 blockade, and many experience immune-related adverse events (irAEs) (2). Moreover, new studies indicate that 5-10% of patients demonstrate accelerated cancer progression after anti-PD-1 treatment, contrary to predicted responses based on current mechanistic models (3). Given such significant successes and failures, a better understanding of how to target the PD-1 signaling pathway is needed. Understanding the underlying mechanisms of clinical responses will promote development of more nuanced pathway-based therapeutics.

A recent publication summarized that 4,062 out of 4,897 ongoing immunotherapy trials are testing PD-1 inhibition in combination regimens with other immunotherapies, targeted therapies, chemotherapies, and radiotherapies (1). Of these combination approaches, immunotherapies lead the space with 1,058 trials, and targeted therapies closely follow with 1,008 trials. The number of monotherapy trials continues to decrease, with less than 20% of active trials using monotherapies against PD-1 or PD-L1, a trend consistent with previous updates (4).

**Figure 1:**
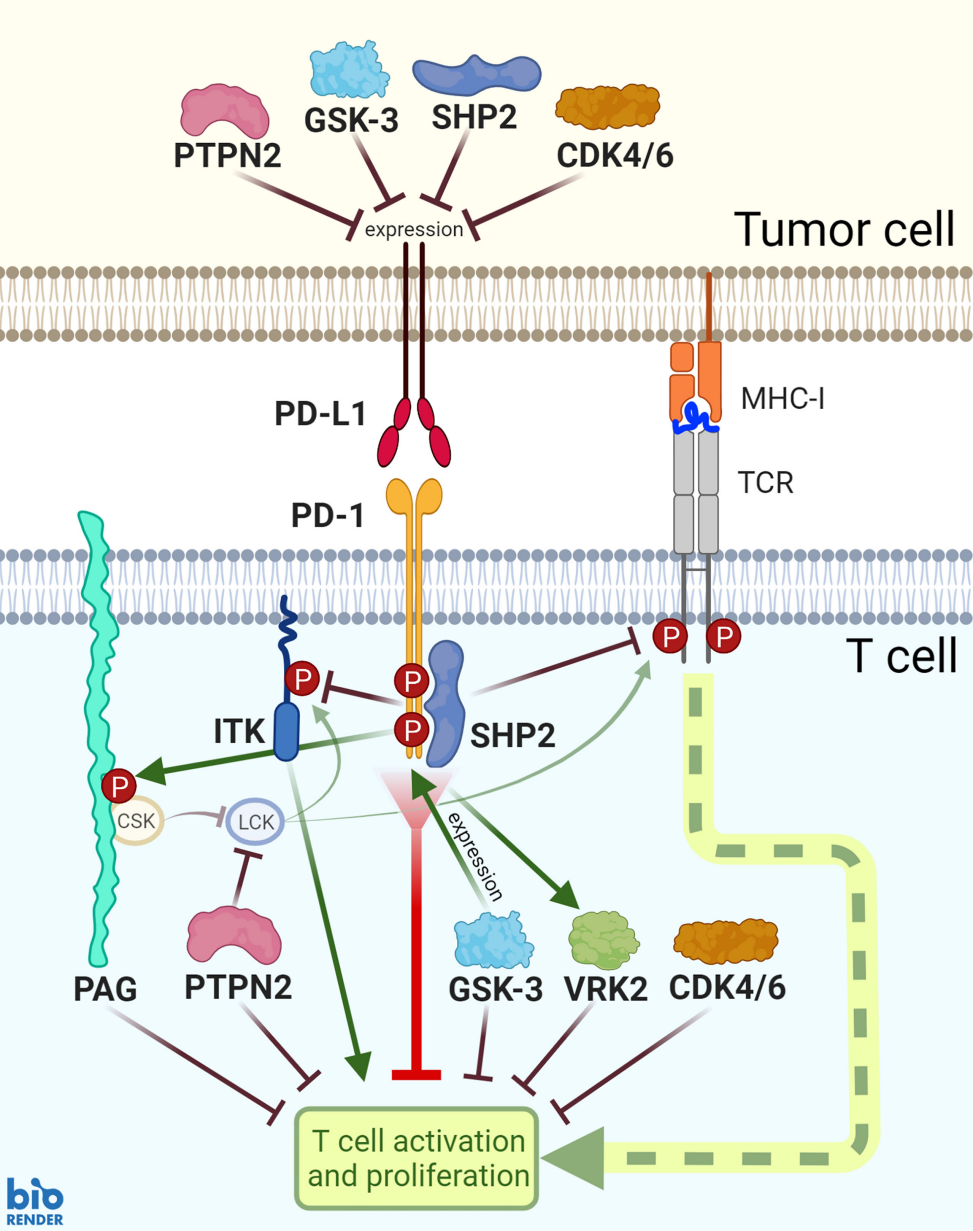
PD-1 functions and PD-L1 expression are mediated by SHP2, PTPN2, PAG, ITK, GSK-3, VRK2, and CDK4/6 signaling. SHP2 is recruited downstream of PD-1 ligation and mediates a number of subsequent signaling events. Additionally, PD-1 ligation is associated with enhanced activity of inhibitory proteins PAG and VRK2 and inhibition of ITK. GSK-3 activates transcription factors to induce PD-1 expression; while GSK-3, PTPN2, SHP2, and CDK4/6 inhibit PD-L1 expression.

As anti-PD-1 clinical trials continue to move towards combination strategies, we describe in this work effector proteins that are associated with PD-1 downstream signaling and function in T cells: SHP2, ITK, VRK2, PTPN2, GSK-3, CDK4/6, and PAG (**Figure 1**). Through exploring their complex mechanistic involvement in T cell anti-tumor responses, we analyze their promise as therapeutic targets in combination with PD-1 blockade. As many of the potential targets are also expressed in tumor cells, we also consider the therapeutic impact within the tumor cell, but mainly focus on the promise of logically designed T cell intrinsic combination approaches.

## PD-1 Signaling

To become fully activated, T cells require at least two signals: engagement of the T cell receptor (TCR) by peptide-loaded major histocompatibility complex (MHC), and co-receptor interactions with ligands on antigen-presenting cells (APC) (5). T cells display a dynamic and complex network of stimulatory and inhibitory co-receptors. Among these, inhibitory receptors provide signals that terminate immune responses and restore homeostasis (6). We discovered that the mechanism by which the co-receptors CTLA-4 and CD28 modulate T cell adhesion is through recruitment of calcium-promoted Ras inactivator (CAPRI) (7). We also reported that engagement of the inhibitory receptor PD-1 by its ligands inhibits Rap1 activation and LFA-1-mediated adhesion (8).

PD-1 is a monomeric transmembrane protein consisting of 288 amino acids, with a single extracellular IgV domain, followed by a transmembrane region and a cytoplasmic tail (9). Despite extensive use of anti-PD-1 antibodies in the clinic, available data on the signaling pathways downstream of this receptor are limited. Because PD-1 does not have direct enzymatic function, it instead serves as a scaffold for other proteins that mediate downstream inhibitory functions (10). Following T cell antigen recognition, PD-1 surface expression is increased, allowing it to bind to its ligands, programmed death ligand 1 (PD-L1) and programmed death ligand 2 (PD-L2) (11). PD-1 then recruits SH2-containing tyrosine phosphatase SHP2. Through mutational analysis of the PD-1 cytoplasmic tail, it has been shown that phosphorylation of the tyrosine within the immunoreceptor tyrosine-based switch motif (ITSM; TXpYXXV/I) following ligand binding recruits SHP2 (12–14); however, for full enzymatic activity, SHP2 must unfold into its active conformation. This is enhanced by phosphorylation of PD-1’s immunoreceptor tyrosine-based inhibitory motif (ITIM; V/L/I/XpYXX/L/V) (13).

After PD-1 binds PD-L1 or PD-L2 and recruits and activates SHP2, this phosphatase then dephosphorylates proteins critical for TCR signaling, such as CD3, ZAP70, and C3G (15). Despite the structural similarity between SHP1 and SHP2, only the latter is recruited to the tail of PD-1 (13). Further, the full mechanism connecting PD-1 engagement with SHP2 enzymatic activation remains largely unknown, and is an important area of ongoing research, as is identification of the PD-1 associated substrates of SHP2 (16, 17).

## SHP2

Src homology domain-2 (SH2)-containing protein tyrosine phosphatase 2 (SHP2; PTPN11) is a non-receptor tyrosine phosphatase that is expressed in the cytoplasm of cells throughout the body (18). The SHP2 protein is composed of two tandem SH2 domains, a protein tyrosine phosphatase (PTP) domain, and a hydrophobic tail with two tyrosine phosphorylation sites (19). In the inactive state, the proximal SH2 domain is folded onto the PTP site in a closed, autoinhibitory conformation. SHP2 binds to its targets with its SH2 domains, and engagement of both SH2 domains results in highest enzymatic function (20).

SHP2 is recruited to many signaling cascades and plays a role in diverse functions, from proliferation to migration (21). It also plays a role in many diseases, with mutations associated with Noonan syndrome, LEOPARD syndrome, and childhood leukemia (22, 23). Beyond this, SHP2 is implicated in many essential pathways of cancer cells, including proliferation, metastasis, and drug resistance. As such, it has been linked to many gastrointestinal, respiratory, blood, and other cancers, and has drawn attention as a potential therapeutic target (24).

### T Cells

SHP2 is recruited to many important signaling cascades, including those downstream of TCR and PD-1 ligation. The signaling pathways downstream of PD-1 that result in the inhibition of T cell functions remain poorly understood (25), but the need for SHP2 in mediating PD-1 function is perhaps the most well-known aspect of PD-1 biology. SHP2 is thought to mediate PD-1 inhibition of T cell function by dephosphorylating tyrosines within the TCR complex to inhibit downstream cascades, including the co-stimulatory receptor CD28 (26). However, SHP2 also plays a critical – and possibly opposing – role in supporting TCR-mediated T cell activation. In this context, SHP2 is considered a positive regulator of T cell activation, using dephosphorylation to turn on positive T-cell regulators (e.g. AKT, ERK) and turn off negative T cell regulators (e.g. CSK, CRK and PAG) (27–30). This paradigm identifies the same SHP2 enzyme serving as a key mediator of two pathways that have opposing functions. There are multiple proposed models explaining how this is executed within the cell. The temporal segregation model proposes that because PD-1 expression is induced only after activation of the TCR, SHP2 first acts downstream of the TCR during the early phase of T cell activation, and transitions to the PD-1 pathway at a later stage of T cell activation after PD-1 expression is increased (31). An alternative model is differential substrate targeting. This model explains that SHP2 has different targets downstream of the TCR and PD-1, and in this way mediates different cellular functions (28, 32). To better resolve the complexity between TCR and PD-1 signalosomes, we need to continue efforts to determine both shared and unique molecular partners and signaling mechanisms involving SHP2, and to identify SHP2 substrates within each of these pathways. The answers to these questions have the potential for significant clinical impact as they may explain the confounding results coming out of clinical trials with SHP2 inhibitors.

### Therapeutic Targeting

New promise for therapeutic targeting of SHP2 may lie in combined inhibition of SHP2 and PD-1. A study of methylene blue, a chemical with FDA approval for the treatment of methemoglobinemia, found that it inhibits PD-1 signaling by interfering with SHP2 binding, and interferes with tumor allograft growth (33). Importantly, this work demonstrates that interfering with the mediators of PD-1 signaling can have a favorable impact on tumor progression, and is a valid therapeutic strategy.

Interestingly, it is the activity of SHP2, rather than its expression level, that contributes to the expression of PD-L1 on tumor cells (34). In turn, the expression of PD-L1 correlates with tumor response to immune checkpoint inhibitors in non-small cell lung cancer (NSCLC) (34). This suggests that current allosteric inhibitors of SHP2 activity may enhance anti-PD-1 efficacy. The fact that a majority of patients with NSCLC do not respond to immune checkpoint inhibitor monotherapy makes it an attractive model for the study of these combinations (35). In a mouse model of anti-PD-1-resistant NSCLC, the triple therapy of anti-PD-1, the oral SHP2 inhibitor SHP099, and radiotherapy had strong anti-tumor effects (36). Similarly, the combined administration of anti-PD-1 antibody and SHP099 had a greater anti-tumor effect than either therapy individually in two murine colon cancer xenograft models (37).

The contribution of SHP2 to PD-1 signaling in T cells was most directly studied in a T cell confined SHP2 knockout (KO) mouse study that found no effect on controlling tumor growth or on the efficacy of PD-1 antibodies, likely due to the impact on SHP2’s role in both T cell activating and inhibitory pathways. The T cell SHP2 KO also did not impact the efficacy of PD-1 antibodies, implying that alternative phosphatases may be recruited to PD-1 in the absence of SHP2 (38). Importantly, this leaves open the potential for an additive effect of SHP2- and PD-1- targeting combination therapy strategies.

There are currently 30 trials on ClinicalTrials.gov testing SHP2 inhibitors in cancer patients. Within these trials, there are 11 SHP2 inhibitors being tested, most commonly TNO155, and sodium stibogluconate (a drug primarily used for Leishmaniasis). Five trials are testing SHP2 inhibitors in combination with anti-PD-1 biologics, all of which are still recruiting and have no available results.

SHP2 is an exciting therapeutic target for combination strategies in cancer. SHP2 is known to be pro-oncogenic in many cancers and simultaneously involved in the inhibitory PD-1 pathway in T cells. Thus, intervention with SHP2 inhibitors might concomitantly inhibit cancer cells and activate the anti-tumor immune response. Combination with anti-PD-1/PD-L1 biologic agents is a particularly intriguing area of work since they have overlapping but non-redundant functions. Better yet, SHP2 inhibitors *in vivo* enhance response to anti-PD-1 therapy through PD-L1 upregulation. This would expand the percentage of patients that may respond to anti-PD-1 therapeutic mechanisms. Further, since SHP2 also plays a role in T cell activation, SHP2 inhibitors might help avoid immune related adverse events seen with anti-PD-1 antibody therapy.

## ITK

IL-2 inducible T cell kinase (ITK) is a member of the TEC family of kinases with particular importance in T cells. The other members of the protein family are Tec, BTK, BMX, and RLK (39). All TEC kinases include a Tec homology (TH) domain with a zinc binding region and proline rich regions. From N to C termini, ITK includes an N-terminal PH domain, a TH domain, and three SH catalytic domains (39). Unlike other family members, ITK is expressed only in T cells, NK cells, NKT cells, and mast cells (40, 41). ITK deficiency results in susceptibility to severe infections with Epstein Barr virus (EBV) (42).

### Tumor Cells

ITK is highly expressed not just in normal T cells, but also in T cell associated malignancies (43). Genetic and pharmacological inhibition of ITK compromises the proliferation, adhesion, invasion, and migration of malignant T cells, which position this kinase as a target for the treatment of primary T cell tumors (43). The promise of targeting ITK in cancer is bolstered by the growing success of targeting protein family member BTK, which plays a similar role in B cells and B cell tumors. BTK has been found to regulate cell proliferation, survival, and migration in various B cell malignancies. Targeting BTK with recently developed BTK inhibitors has been approved by the FDA to treat several B cell malignancies (44). Recent studies have established also that BTK is expressed and plays pro-tumorigenic roles in several epithelial cancers (45).

### T Cells

ITK plays a modulatory role in TCR signaling. Unlike ZAP70 and LCK, ITK is not an obligate component of the TCR cascade. Instead, ITK functions as a fine-tuning dial, to translate variations in TCR signal strength into differential programs of gene expression (46). Upon T cell activation, a series of signaling events lead to the recruitment of ITK to the cell membrane in the vicinity of the primed TCR, where it is phosphorylated by LCK on Tyr 512. This leads to ITK autophosphorylation of Tyr 180 and to subsequent downstream phosphorylation of PLCγ1 and LAT, and NFAT translocation into the nucleus (47). Consequently, it was shown *in vivo* that ITK is not required for TCR signaling (48). In the absence of ITK, some aspects of T cell activation appear normal, whereas other T cell functions are impaired. Further, studies in ITK knockout mice show that T cell function is impaired but not entirely blocked (49, 50), a finding that is consistent with a modulating role for ITK, rather than an all-or-nothing molecular switch.

In addition to its role in TCR signaling, ITK is also an important kinase in the PD-1 pathway. Through a phosphoproteomic study, we found ITK mediates many phosphorylation events downstream of PD-1 ligation (10). Using genetic and pharmacological approaches, we then discovered that SHP2 dephosphorylates ITK specifically downstream of PD-1 and that this event is associated with PD-1 function (17). Notably, SHP2 only dephosphorylates ITK in its role downstream of PD-1 signaling. Since ITK is a SHP2-dependent specific mediator of PD-1 signaling, the combination of ITK inhibitors with PD-1 blockade may improve upon PD-1 monotherapy in the treatment of cancer.

### Therapeutic Targeting

Ibrutinib is an approved therapy for B cell malignancies that covalently inhibits both BTK and ITK (51). In a recent study, blood samples collected from leukemia patients treated with ibrutinib monotherapy showed downregulated PD-L1 expression on the leukemic cells. Further, the same analysis showed that this was mediated through inhibition of STAT3. Similarly, downregulation of PD-1 expression was observed in the CD4 and CD8 T cells. Taken together, these findings provide the mechanistic basis for immunomodulation by ibrutinib through inhibition of the STAT3 pathway, a critical pathway in inducing and sustaining tumor immune tolerance. This data also merits testing of combination treatments combining ibrutinib with immune checkpoint inhibitors (52).

Indeed, a published study reported that the combination of anti-PD-L1 antibody and ibrutinib suppressed tumor growth in mouse models of lymphoma that were intrinsically insensitive to ibrutinib (53). The combined effect of these two agents was also documented in mice models of triple negative breast cancer and colon cancer. The enhanced therapeutic activity of PD-L1 blockade by ibrutinib was accompanied by enhanced anti-tumor T cell immune responses. This study suggested that the combination of PD-1 or PD-L1 blockade and ibrutinib should be tested in the clinic for the therapy not only of lymphoma but also in solid tumors that do not even express BTK or ITK (53). Similarly, a study using the Eµ-TCL1 adoptive transfer mouse model of chronic lymphocytic leukemia (CLL), observed that combination of ibrutinib with blocking antibodies targeting the PD-1 or PD-L1 axis *in vivo* improved CD8 T cell effector function and control of lymphocyte proliferation (54). This study suggested that the strong immunomodulatory effects of ibrutinib and its combination with immune checkpoint blockade was a promising approach to treat CLL (54).

Regulatory T cells (Tregs) play an important role in controlling autoimmunity and limiting tissue damage and inflammation. It was shown that either genetic ablation of ITK or inhibition of ITK pharmacologically results in increased number of Tregs (55, 56). This was shown to avert the formation of acute graft-versus-host disease *in vivo*, in part by reducing T cell proliferation and cytokine production. More interestingly, disrupting the SLP76—ITK interaction using a specific peptide inhibitor led to enhanced Treg development in both mouse and primary human cells. Thus, it was suggested that targeting ITK could be a therapeutic strategy to treat not just autoimmunity, but also immune related toxicity of PD-1 blockade (57). Altogether, while additional studies are needed to clarify the impact of treating cancer with a combination of PD-1 blockade and ITK/BTK inhibitors, this possibility is mechanistically promising and clinically feasible with current approved drugs.

## VRK2

Vaccinia-related kinase 2 (VRK2) is a serine/threonine kinase that in humans is encoded by the VRK2 gene (58). It is a member of the VRK family, which includes VRK1, VRK2, and VRK3 (59). VRK2 is widely expressed in human tissues and has increased expression in actively dividing cells, such as leukocytes and carcinomas (60). VRK2 has two splice forms, VRK2A and VRK2B. The VRK2A isoform is much more common, and includes a C-terminal hydrophobic tail that tethers it to organelles like the endoplasmic reticulum and mitochondria (61). The rarer isoform, VRK2B, lacks a hydrophobic tail and is found in the nucleus and cytoplasm (61). Among other targets, VRK2 modulates several MAPK signaling pathways through target phosphorylation and impacting the composition of signaling complexes (62).

### Tumor Cells

VRK2 is most highly expressed in cells undergoing division, and is therefore present in notable amounts in some cancer cells (63). High VRK2 expression levels are associated with unfavorable prognosis in renal, liver, and pancreatic cancers (63, 64). In pancreatic cancer, VRK2 phosphorylates and stabilizes cell cycle kinase Plk1, resulting in Plk1 overexpression to facilitate pancreatic cancer proliferation and chemotherapy resistance (65). Similarly, pediatric and adult gliomas and neuroblastomas require either VRK1 or VRK2, which have overlapping but essential pro-survival function in these cancers (66). In breast cancer, VRK2 has been found to facilitate tumor cell invasion through phosphorylating transcription factor NFAT1 to increased COX2 expression (60). COX2 is associated with invasive breast cancer, metastasis, and poor prognosis (67, 68). VRK2 is also thought to be protective against apoptosis (69). In contrast, another study found that low VRK2 levels are associated with the abnormal MEK/ERK signaling seen in breast cancer; thus, VRK2 has a complex signaling role in cancer (70).

### T Cells

VRK2 has only recently become the subject of study in T cells. Downstream of both TCR and PD-1 ligation, PAK2, a mediator cytoskeleton reorganization, is phosphorylated by VRK2 (71). Thus, VRK2 and PAK2 have conflicting roles downstream of TCR activating signals versus PD-1 inactivating signals; this is analogous to what is known about SHP2 (71). Within the PD-1 pathway, VRK2 mediates one quarter of all the phosphorylation events downstream of PD-1 ligation. In fact, lack of VRK2 activity inhibits PD-1 function, both *in vitro* and *in vivo* (71). The phenotype of VRK2 KO mice is similar to PD-1 KO mice, both presenting with lymphoproliferation and activated T cell subsets. Additionally, in an MC38 murine tumor model, a VRK2 inhibitor AZD-7762 decreased tumor growth in a VRK2-dependent and T cell-dependent manner. When AZD-7762 was used in combination with anti-PD-1 therapy, the mice showed an additive therapeutic impact in terms of tumor growth and final tumor volume (71). Thus, VRK2 acts as an inhibitory kinase that mediates the functions of PD-1 *in vivo*. The fact that a kinase, and not just the phosphatase SHP2, mediates PD-1 function is not just exciting, but also offers opportunities to develop novel kinase inhibitors as an alternative to checkpoint blockades. Though expression of VRK2 is required for PD-1 function, VRK2 has two domains, a kinase domain and a protein-protein docking region, and the contribution of each of the domains to its functions downstream of PD-1 is not completely understood. This knowledge is much needed for better understanding PD-1 signaling and to allow design of optimal VRK2 inhibitors.

### Therapeutic Targeting

Because VRK family kinases have a different ATP binding domain structure than other kinases, they are resistant to most current kinase inhibitors. However, studies have noted which inhibitors VRK family proteins are most sensitive to, which is useful in preclinical studies since high drug concentrations are necessary (58). More importantly, work in 2019 began the development of aminopyridine-based compounds to specifically inhibit VRK1 and VRK2 (72). Fortunately, the unique structure of these kinases means future inhibitors will be specific to VRK family proteins, with little unintended binding to other kinases, making VRK2 a very attractive drug target.

Since VRK2 plays both a pro-tumor role in malignant cells and an inhibitory role in T cells, it has poignant therapeutic potential in cancer. Further, since VRK2 mediates only a subset of PD-1 signaling and also participates in TCR signaling, it has the potential to improve response rates and/or decrease immune related adverse events when used in combination with anti-PD-1 agents, compared to immune checkpoint inhibitor therapy alone.

## PTPN2

Protein tyrosine phosphatase non-receptor type 2 (PTPN2, or TCPTP) is a ubiquitously expressed regulator of inflammation. PTPN2 is known to dephosphorylate tyrosine kinases, including JAK1/2/3, SRC family kinases, and STAT1/3/5/6. PTPN2 inhibits pro-inflammatory pathways, including IFN-γ signaling, and mutations in PTPN2 are associated with chronic inflammatory and autoimmune diseases, including type I diabetes and Crohn’s disease (73). In contrast, high PTPN2 function is associated with several cancers. PTPN2 levels are high in some gliomas, laryngocarcinoma, and thyroid cancer, with high PTPN2 levels in cancer cells under oxidative stress and inflammatory conditions (74–77). PTPN2 also plays the role of an oncogene in colon cancer by inhibiting the inflammasome (78, 79). In contrast, PTPN2 is likely a tumor suppressor in acute lymphoblastic leukemia (ALL) because it inhibits JAK1, which is oncogenic in ALL, though PTPN2 levels are often low in ALL (80, 81).

### T Cells

PTPN2 plays an important role in the development and activation of T cells. PTPN2 knockout mice all died by five weeks of age but showed apparently normal development of CD4 and CD8 T cells (82). However, it is known that PTPN2 dephosphorylates STAT5 in the nucleus to facilitate the transition of precursor cells through the DN2 and DN3 thymocyte differentiation steps to become mature T cells (83). PTPN2 also negatively regulates LCK which, along with STAT5, helps control T cell commitment to alpha/beta versus gamma/delta TCR expression (83). PTPN2 also helps control the T cell activation threshold by dephosphorylating SRC family kinases necessary for functions downstream of TCR ligation (73, 84). Studies *in vitro* and *in vivo* in mice have shown that deletion of PTPN2 in T cells results in enhanced CD8 T cell proliferation and survival, with decreased dependency on pro-survival cytokines like IL-2 and IL-15 (85).

Additionally, PTPN2 is critical for maintaining peripheral tolerance to self-antigens cross-presented on dendritic cells (86). PTPN2 loss of function (LOF) results in systemic autoinflammation in mice, with high systemic cytokines and anti-nuclear antibody levels, supporting the association between PTPN2 LOF and several autoimmune conditions including type I diabetes, Crohn’s disease, and Celiac disease (73, 86, 87). PTPN2 has also been shown to play an important role in T cell exhaustion, a state in which chronically stimulated T cells lose their ability to target cancer cells or chronic infections. PTPN2 also reduces type I interferon signaling, leading to a terminally exhausted T cell state. Loss of PTPN2 allows T cells to expand in response to re-stimulation (85). Along with increased proliferation, PTPN2 deficient mice also have enhanced cytotoxicity among Tim-3^+^ cells (a marker of the terminally exhausted state), and augmented anti-tumor function, tumor control, and anti-PD-1 responses (88).

### Tumor Cells

A CRISPR-Cas9 screen identified PTPN2 deletion to enhance response to anti-PD-1 immunotherapy in a murine tumor model with B16 melanoma cells (89). The PTPN2-null B16 cells had enhanced antigen presentation and increased susceptibility to cytotoxic CD8 T cells (89). PTPN2-null tumors had enhanced CD8 infiltration, and impaired growth in response to IFN-γ (89). An intact JAK/STAT pathway downstream of the IFN-γ receptor on tumor cells induces PD-L1 expression and is critical for response to anti-PD-1 therapy. Since PTPN2 is a negative regulator of JAK/STAT signaling, inhibiting PTPN2 predictably increases responses to anti-PD-L1 therapy in murine melanoma YUMM1.1 cells *in vitro* and *in vivo* (90).

### Therapeutic Targeting

Though targeting a phosphatase will include the challenge of developing an inhibitor with appropriate specificity, PTPN2 has proven a promising target. In one study, PTPN2 was targeted by a compound composed of copper-sulfate nano-photothermal materials carrying Cas9 and oligonucleotides to generate a mutation in PTPN2. Its use in mice caused tumor hyperthermia, PTPN2 depletion, increased T cell infiltration into the tumor, and higher intratumoral IFN-γ and TNF-α levels (91). Controlled enhancement of PTPN2 function may be beneficial in autoimmune disease, while targeted inhibition of PTPN2 may help enhance the immune response to cancer. Based on studies with B16 melanoma cells, which typically show resistance to PD-1 therapy, adding a PTPN2 inhibitor to anti-PD-1 regimens may help expand the pool of cancer patients responsive to checkpoint inhibition (88). Yet, because of PTPN2’s role in maintaining peripheral tolerance, future therapeutic efforts should consider tumor microenvironment specific targeting and be cognizant of immune related adverse events.

## GSK-3

Glycogen synthase kinase 3 (GSK-3) is a ubiquitously expressed serine/threonine kinase and protooncogene (92, 93). GSK-3 has two isoforms, GSK-3α and GSK-3β, which have homologous kinase domains but divergent C-terminal domains, as well as non-redundant functionality (94). GSK-3 activity is controlled by phosphorylation: phosphorylation at Ser 9 inactivates GSK-3β (Ser 21 in GSK-3α), while phosphorylation at Tyr 216 increases GSK-3β activity (Tyr 279 in GSK-3α) (94). With over 100 downstream phosphorylation targets including transcription factors β-catenin, NFκB, NFAT, CREB, c-Jun, and AP1 (92, 95), GSK-3 has been shown to play a role in many cell functions including glycogen and protein metabolism, tumor growth, metastasis, and various immune functions (92, 96–98).

### Tumor Cells

GSK-3 has clear pro-tumor actions in many cancers, notably including KRAS-mutant tumors (92). For example, in non-small cell lung cancer (NSCLC), GSK-3β expression is associated with cervical lymph node metastases, poor differentiation, advanced stage, late diagnosis, and worse survival, while inhibiting GSK-3 can result in cancer cell apoptosis and cell cycle arrest (99, 100). *In vivo* models of pancreatic cancer demonstrate an inverse relationship between survival and the nuclear amount of aberrant GSK-3 (93). GSK-3 inhibition in mice by knockout or small molecule inhibitors increases cytotoxicity against viral infections and tumor cell models of gastric cancer (MFC), melanoma (B16), lymphoma (EL-4), colon cancer (MC38), colorectal cancer (CT26), pancreatic cancer (KPC), and lung cancer (LLC) (94, 101–104).

### T Cells

Uniquely, GSK-3 is found in the active state in resting T cells. When active, GSK-3 inhibits T cell proliferation and IL-2 production (105, 106). T cell activation through TCR and CD28 ligation results in PI3K/AKT signaling. This pathway phosphorylates and inactivates GSK-3, which increases T-bet expression levels (92). Inactivation of GSK-3 has been shown to be crucial for T cell activation. In fact, GSK-3 inhibition can substitute for CD28 signaling to induce co-stimulation of T cell proliferation (107, 108). Additionally, GSK-3 inhibition with small molecule inhibitor TWS119 has been shown to induce the Wnt/β-catenin pathway to revert CD8 memory T cells into cytotoxic progenitor memory stem cells that can undergo self-renewal (109).

Notably, GSK-3 was also identified as a major regulator of checkpoint protein expression (110). Cells with GSK-3 inhibition through siRNA or small molecule inhibitors show increased T-bet expression in response to TCR stimulation, which inhibits transcription of both PD-1 in CD8 T cells and Tregs, and LAG3 in CD4 and CD8 T cells (101, 111, 112). Consistently, GSK-3β knockout or *in vivo* inhibition in mice results in decreased PD-1 expression in CD8 T cells; increased expression of T-bet, granzyme B, and IFN-γ; enhanced CTL function ex vivo; increased tumor infiltration; and reverted T cell exhaustion in an LCMV model (94, 101, 113). Treatment of CAR-T cells with GSK-3 inhibitors during T cell activation resulted in lower PD-1 levels. These cells showed increased proliferation, decreased exhaustion, and full tumor elimination in a GBM mouse tumor model (114). GSK-3 inhibition also decreases T cell motility and number of cell-cell contacts, but this is overpowered by greatly enhanced cytotoxicity (115).

### Therapeutic Targeting

Given its important role in immune function, GSK-3 inhibitors have been tested for their impact on anti-tumor immunity. Preclinical studies found that GSK-3 inhibitors are as effective as anti-PD-1 or anti-PD-L1 antibodies to inhibit tumor growth in mice (101, 103). More importantly, anti-PD-1 and GSK-3 inhibitor combinations may be effective to treat solid tumors that are otherwise unresponsive to immune checkpoint blockade. This is likely because, GSK-3β phosphorylates PD-L1 in tumor cells to induce its degradation, and GSK-3β inactivation can be seen in some cancers to stabilize PD-L1 expression (116, 117). Similarly, inhibiting GSK-3β with the chemotherapy-sensitization combination disulfiram and copper stabilizes PD-L1 expression in a hepatocellular carcinoma model (118). Compared to anti-PD-1 alone, combination therapy with anti-PD-1 plus GSK-3 inhibitors increased the ratio of CD8 effector memory cells to CD4 Tregs within the tumor (119). Additionally, tumor growth is further inhibited when anti-LAG3 antibodies and GSK-3 inhibitors are used in combination in mice. More specifically, anti-LAG3 and GSK-3 inhibitor SB415286 decreased tumor growth and prevented lung metastasis in a murine melanoma model (112). Anti-PD-1 and anti-LAG3 are a promising combination therapy (120), yet in this study, the combination of a GSK-3 inhibitor and anti-LAG3 showed even stronger therapeutic efficacy (112).

GSK-3 inhibitors are particularly promising for their potential ability to both directly inhibit malignant cells and also enhance the immune response (92). Following a number of promising pre-clinical results, there are eighteen clinical trials completed or ongoing using GSK-3 inhibitors as therapy against a wide range of cancers. Several clinical trials do not give a GSK-3 inhibitor directly to the patients, but rather pre-treat NK cells or CAR-T cells with GSK-3 inhibitors to enhance the anti-tumor activity of these lymphocytes for cellular immunotherapy. During generation of anti-CD19-CAR-T cells, culture conditions include IL-21, IL-7, and GSK-3 inhibitor TWS119. Three of the ten patients in this phase I clinical trial showed regression of their B-cell malignancy, and toxicities were mild and did not include graft-versus-host disease (121). NK studies began with ex vivo experiments showing that peripheral NK cells cultured with IL-15 + GSK-3 inhibitor CHIR99021 upregulate CD57 and undergo late-stage maturation into a maximally cytotoxic form (122, 123). CHIR99021-treated NK cells have increased production of TNF-α and IFN-γ and enhanced antibody dependent cellular cytotoxicity (ADCC) *in vitro*. Adoptive cell transfer of these cells into mice resulted in stronger/prolonged control against acute myeloid leukemia and ovarian cancer models (122, 124). From these results, three Phase I clinical trials are underway that use CHIR99021-treated NK cells with IL-2 or chemotherapy for patients with lymphoma or a number of solid organ tumors.

These results suggest that GSK-3 inhibitors may serve as a promising addition to cancer therapeutic strategies. GSK-3 inhibition lacks harmful effects on normal cell and organ function in rodent studies and has long history of safe use in bipolar disorder (lithium carbonate inhibits GSK-3β activity) (92, 96). Preclinical studies also suggest that GSK-3 inhibitors may help protect against chemotherapy-induced thrombosis and neurotoxicity, and also decrease the development of tolerance to morphine (96). Should GSK-3 combination therapy allow lower dosing of anti-PD-1 agents, there is potential to assuage some anti-PD-1 side effects. With therapeutic mechanisms acting both directly on cancer cells and enhancing immune responses, GSK-3 inhibitors may be an important part of future checkpoint-focused drug combinations.

## CDK4 and CDK6

Cyclin dependent kinase (CDK) 4 and CDK6 are important kinases in the cell cycle. CDK4/6 activity is regulated by tightly controlled levels of cyclin D (125, 126). Cyclin D binds and activates CDK4/6 during G1 of the cell cycle, and together they phosphorylate retinoblastoma protein (RB1) to promote cell cycle progression into S phase through transcription of genes controlled by transcription factor E2F (127). Cyclin D1 and CDK4/6 promote cell cycle progression and prevent cell senescence through activation of transcription factor FOXM1, inactivation of TGFβ-mediator SMAD3, and indirect activation of p53 (128–130). Constitutive activation of the complex of cyclin D and CDK4/6 results in uncontrolled cell proliferation, and has a strong link to many cancers (127).

### Tumor Cells

Cyclin D1 is often genetically upregulated *via* chromosomal translocation in mantle-cell lymphoma, multiple myeloma, and plasma cell leukemia, as well as a significant fraction of breast cancers, head and neck, and esophageal squamous cell carcinomas (131, 132). Other cancers overexpress cyclin D1/2/3, CDK4/6, or have lower levels of CDK4/6 inhibitors (127). Mouse studies have shown that overexpression of cyclin D or CDK4/6 increases susceptibility to breast cancer, while ablation induces tumor shrinkage in HER2+ and NSCLC tumor models (133–135). Due to its evident role in proliferation and cancer, several CDK4/6 inhibitors have been generated, including palbociclib and ribociclib, which specifically target CDK4/6, and abemaciclib which targets CDK4/6 and other similar kinases (127). These agents likely directly inhibit CDK4/6, perhaps by preventing the formation of cyclin D-CDK4/6 complexes or potentially decreasing their stability, though lower complex levels are not seen with inhibitor use (136, 137). Abemaciclib most efficiently crosses the blood brain barrier, and effectiveness of these drugs is best predicted by intact RB1 expression in the tumor cells (138, 139). These and other CDK4/6 inhibitors are currently being used in hundreds of clinical trials across a variety of cancer types. Due to results showing increased progression-free and overall survival, palbociclib, ribociclib, and abemaciclib have all been approved for treatment of advanced or metastatic hormone receptor positive breast cancers (127).

### T Cells

In addition to impacting the cancer cell, CDK4/6 inhibitors also impact the immune response. CDK4/6 inhibitors help turn “cold” tumors into “hot” tumors. Treatment in mice induced tumor cell release of type III interferons and increased MHC antigen presentation (140). Increased levels of chemokines such as CXCL9 and CXCL10 from CDK4/6 inhibition also drive T cell tumor infiltration (141). Inhibiting CDK4/6 also reduces Treg proliferation to decrease the intra-tumoral Treg/CTL ratio (140). Additionally, CDK4/6 inhibition enhances transcription of genes under control of the transcription factor NFAT. Without CDK4/6 phosphorylation of NFAT, nuclear NFAT levels increase to promote transcription of T cell activating proteins (141). Abemaciclib treatment was specifically found to increase expression of immune checkpoint proteins on T cells, including CD137, PD-L1 and TIM3 (142). Interestingly, regardless of RB1 status, CDK4/6 inhibitors increase PD-L1 expression on tumor cells by decreasing its rate of degradation (143). Consequently, the combination of CDK4/6 inhibitors with anti-PD-1/PD-L1 therapy showed an additive effect in animal tumor models (142). Different studies showed greater benefit with a varied schedule of drug administration, for example delaying the start of anti-PD-L1 drugs until after the start of abemaciclib or vice versa (142, 144). This treatment regimen resulted in enhanced T cell tumor infiltration, increased MHC-I/II expression on tumor cells and APCs, and improved memory formation (142). Further, a combination of a CDK4/6 inhibitor and a PI3K inhibitor significantly improved response to anti-PD-1 or anti-CTLA-4 therapy (145). Uniquely, CDK4/6 inhibitor trilaciclib has been shown to protect normal cells from chemo-related cytotoxicity, including preserving hematopoietic stem cells to decrease myelosuppression, and thus may also be an important contribution to combination therapy (146, 147).

### Therapeutic Targeting

Notably, preclinical trials so far have shown an additive effect of combining CDK4/6 inhibitor with checkpoint blockade agents, including anti-PD-1 and anti-PD-L1 (144). Since many tumors may harbor intrinsic resistance to immune checkpoint inhibitors or to CDK4/6 inhibitors, these pre-clinical results are quite promising. The use of CDK4/6 inhibitors could have several benefits to clinical response to immune checkpoint therapy (144). These inhibitors likely decrease intrinsic and acquired resistance, and amongst responders, may show an additive response compared to either therapy alone. Further, this effect may be seen even in tumors intrinsically resistant to CDK4/6 inhibitors, as their effect on PD-L1 expression is independent of RB-status in tumor cells. Accordingly, there are nine trials covering many cancer types that combine immune checkpoint agents with CDK4/6 inhibitors; results from these trials may have immense implications on the future of combination therapy.

## PAG

Phosphoprotein associated with glycosphingolipid rich microdomains 1 (PAG) is an inhibitory transmembrane protein that is highly expressed on leukocytes, monocytes, and lymphocytes. PAG has a 16 amino acid extracellular domain and a 397 amino acid cytoplasmic tail with ten tyrosine phosphorylation sites (148). Despite being transmembrane, PAG has no known binding partners (149). PAG is palmitoylated to induce localization within the lipid-rich regions of the membrane, along with many important signaling proteins involved in TCR signal transduction and modification (148, 150–152). Consistently, PAG is recruited to the synapse upon immune synapse formation (153). PAG is a member of the family of transmembrane adaptor proteins (TRAPs) and helps organize signaling through the recruitment of cytosolic kinases and phosphatases (150, 154). PAG also has a C-terminal PDZ domain that binds to EBP50, a protein which connects to actin *via* Ezrin (150, 155–157). PDZ domain proteins are important in immune synapse formation and function (158). Actin is a major cytoskeletal protein essential for many cellular functions, including synapse formation (159). Therefore, PAG likely serves as a link between actin and other lipid-raft proteins important for immune synapse signaling.

### T Cells

In resting T cells, SRC-phosphorylated PAG recruits the inhibitory tyrosine-protein kinase CSK to lipid rich signaling complexes, which results in inactivation of lymphocyte-specific protein tyrosine kinase (LCK) to prevent signaling through the TCR (148, 154, 160, 161). Upon TCR signaling, PAG is dephosphorylated by PTP1B and releases inhibitory CSK for successful TCR signaling (160). CSK is also a negative regulator of c-SRC, a membrane-anchored tyrosine kinase and proto-oncogene (162). PAG also regulates localization of SRC family kinases FYN and LYN, impacting their signaling (163). Thus, PAG is important in regulating the clustering of synapse-related signaling molecules. Multiple studies showed that overexpression of PAG leads to T cell inhibition, while deleting PAG leads to T cell activation. We confirmed these findings and also showed that reduced tumor size in PAG KO mice was associated with increased T cell activity (153). Similarly, Veillette et al. reported recently that T cells from PAG KO mice had increased resistance to T cell anergy (164). PAG KO mice also demonstrate augmented T cell autoimmunity after challenge (such as MOG in EAE model), suggesting that the importance of PAG-mediated negative regulation is apparent under particular types of immune responses (165).

PAG is a mediator of PD-1 signaling. Phosphorylation of PAG is required for the full strength of PD-1 on many T cell effector functions, including cytokine production, adhesion, activation, and TCR signaling. Further, the phosphorylation status of PAG’s many tyrosine phosphorylation sites mediate various PD-1 functions. In the murine MC38 colon adenocarcinoma and B16 melanoma tumor models, our lab showed that PAG KO mice have limited tumor growth and enhanced response to anti-PD-1 treatment (153). This correlated with increased infiltration of both CD4 and CD8 T cells into MC38 tumors in PAG KO mice and enhanced cytotoxicity of PAG KO murine T cells *in vitro* (153). Consistently, patient data shows that high PAG levels are more common among patients who do not respond to anti-PD-1 therapy (153).

### Tumor Cells

Patient data shows that higher expression of wild-type PAG is associated with worse outcomes in many cancers, including breast cancer, cervical squamous cell carcinoma, head and neck squamous cell carcinoma, liver hepatocellular carcinoma, uterine corpus endometrial carcinoma, and lung adenocarcinoma (153, 166). High PAG expression also induces resistance to radiotherapy in laryngeal carcinoma (167, 168).

### Therapeutic Targeting

Therapeutic intervention targeting PAG could take the form of an inhibitory antibody or a compound to change expression level. The anti-inflammatory/vasodilatory drug pentoxifylline has been shown to decrease PAG expression levels in T cells (169). Because PAG is a transmembrane protein, it can be targeted by the same approach used in traditional immunotherapy – development of an antibody to bind and inhibit signaling. While all other antibodies in therapeutic use inhibit ligand binding, PAG has no known ligand. Instead, binding PAG with a bulky antibody may displace PAG outside of the narrow immune synapse away from sites of TCR and PD-1 signaling. Because of the importance of the subcellular localization of signaling complexes and the complexity of healthy synapse formation in T cell responses, PAG is a strong candidate for therapeutic targeting. We have generated and begun testing an anti-PAG antibody in murine MC38 and B16 tumor models, with promising results.

As a mediator of PD-1 signaling, PAG serves as a new, perhaps more nuanced target for cancer immunotherapy. Combining anti-PD-1 therapy with antibodies or other compounds targeting PAG will likely further impede inhibitory signaling in T cells, *via* PD-1-related and unrelated mechanisms. Together, this combination therapy might better release T cells to continue targeting chronic antigens, such as in cancer, to improve response rates and avoid PD-1 associated adverse events.

## Conclusions

With the remarkable success of anti-PD-1 therapy limited to a fraction of patients, the field is actively working to identify and test new T cell targets to extend the benefits of immunotherapy to a larger group of cancer patients while reducing chances of immune related adverse events. In response, an increasing number of cancer clinical trials include combinations of checkpoint inhibitors and other immunotherapies, targeted therapies, or other therapeutic techniques (4). Ongoing trials show promise for many cancers that have historically not responded to anti-PD-1/PD-L1 monotherapy. However, the number of current PD-1 combination clinical trials is so staggering that trials are competing for patients (1). One way to thin out the number of unsuccessful future trials is to strive for stronger pre-clinical evidence of a new target’s promise before bringing a new combination strategy to patient trials. This might begin with additional mechanistic and translational studies that elucidate signaling pathways to understand the interplay between two proteins of interest. If we understand the signaling relationship between two proteins within immune cells and target cells, researchers can more logically select target combinations with the most promise to enhance responses and/or decrease side effects. In this review, we highlighted a number of promising kinases, phosphatases, and adaptor proteins with strong mechanistic evidence supporting their use in combination with anti-PD-1/PD-L1 agents.

Combinational approaches could take many forms. A target downstream of PD-1 ligation has the potential to maintain or improve therapeutic effects, or to assuage negative side effects of anti-PD-1 therapy. A new drug along this pathway may amplify or substitute anti-PD-1 in combinational approaches (**Figure 1**). Substitute PD-1-pathway targets would allow pairing with drugs that target alternative complementary pathways. Combination therapy could also be used to increase the PD-L1 expression on tumor cells. Inducing PD-L1 expression increases tumor responsiveness to concurrent or subsequent anti-PD-1/PD-L1 therapy. The signaling of GSK-3, PTPN2, SHP2, and CDK4/6 all decrease PD-L1 expression on tumor cells (**Figure 1**). Thus, if a therapeutic strategy includes an inhibitor against one of these four kinases or phosphatases, it may best be used in combination with an anti-PD-1 agent.

Preclinical trials have already shown an enhanced response to anti-PD-1 agents when used alongside therapeutic inhibition or genetic deletion of all targets discussed here: SHP2, ITK, VRK2, PTPN2, GSK-3, CDK4/6, and PAG (**Table 1**). Yet, additional mechanistic understanding of new targets is essential to avoid unintended side effects including maladaptive impact on the tumor microenvironment. This includes considering the impact on various cell subsets, such as Tregs, tumor immunogenicity, and tumor immune infiltration.

**Table 1:** SHP2, ITK, VRK2, PTPN2, GSK-3, CDK4/6, and PAG all have evidence supporting their relationship to the PD-1 pathway in T cells and pro-tumorigenic role in cancer cells.

	Protein class	Role in PD-1 pathway in T cells ^	Cancer cell intrinsic tumorigenic function ^	Cancer types discussed here	Number of Cancer clinical trials*	Cancer types in clinical trials *	Inhibitors approved or in clinical trial *	Clinical trials with anti-PD-1/PD-L1 *
**SHP2**	Phosphatase	Chemnitz et al., 2004 (27) Sheppard et al., 2004 (32) Fan et al., 2020 (33)	Niihori et al., 2005 (23) Zhang et al., 2015 [review] (24)	Childhood leukemia, GI and respiratory tumors, NSCLC	30	For trials with ICB: NSCLC, head/neck and esophageal SCC, GI stromal cancer, CRC, KRAS mutant solid tumors	TNO155, sodium stibogluconate, RMC-4630, JAB-3312, JAB-3068, RLY-1971, BBP-398, HBI-2376, ERAS-601, SH3809, GDC-1971, ET0038, HS-10381, BPI-442096	NCT04720976, NCT04418661, NCT04721223, NCT04699188, NCT04000529
**ITK**	Kinase	Strazza et al., 2021 (17)	Lechner et al., 2020 [review] (43)	Leukemias, lymphomas, breast cancer (model), colon cancer (model)	> 300	Lymphomas, leukemias, MDS, multiple myeloma, aplastic anemia, RAEB-T, SCLC, CRC, melanoma, head/neck SCC, glioblastoma;, kidney, breast, prostate, gastro-esophageal, lung, and pediatric brain cancers	Ibrutinib, CPI-818	N/A
**VRK2**	Kinase	Peled et al., 2021 (71)	Vazquez-Cedeira et al., 2012 (60, 64–68)	Renal cancer, liver cancer, pancreatic cancer, glioma, neuroblastoma, breast cancer, colon cancer (model)	0	N/A	N/A	N/A
**PTPN2**	Phosphatase	Manguso et al., 2017 (89)	(74–79) TS (80, 81):	Glioma, laryngocarcinoma, thyroid cancer; ALL (TS), melanoma (model)	0	N/A	N/A	N/A
**GSK-3**	Kinase	Steele et al., 2021 (94) Taylor et al., 2016 (101) Taylor et al., 2018 (103) Pokhrel et al., 2021 (111)	Domoto et al., 2020 [review] (96) Alves et al., 2021 (99) Zeng et al., 2014 (100)	KRAS mutant cancers, NSCLC, GBM (model), HCC (model), CRC (model), pancreatic cancer (model), lymphoma (model), melanoma (model), gastric cancer (model)	18	Lymphomas, leukemias, sarcoma, glioma, neuroblastoma, adenoid cystic carcinoma, meningioma, SCLC, CRC, neuroendocrine tumor; pancreatic, renal, bone, breast, lung, salivary gland, esophageal, prostate, thyroid, and stomach cancers	TWS119, lithium carbonate, CHIR99021, 9-ING-41, LY2090314	N/A
**CDK4** **CDK6**	Kinase	Schaer et al., 2018 (142) Jerby-Arnon et al., 2018 (144)	Wang et al., 1994 (133) Landis et al., 2006 (134) Puyol et al., 2010 (135)	Mantle-cell lymphoma, multiple myeloma, plasma cell leukemia, breast cancer, head/neck and esophageal SCC	> 300	For trials with ICB: melanoma, pancreatic cancer, breast cancer, head/neck SCC, NSCLC, mesothelioma, liposarcoma, GI cancers	Ribociclib, palbociclib, abemaciclib, Trilaciclib, lerociclib, SHR6390, PF-06873600, FNC-437, Birociclib, HS-10342, CS3002	NCT02791334, NCT03292250, NCT03386929, NCT03654833, NCT03805399, NCT04213404, NCT04360941, NCT04438824, NCT05139082
**PAG**	Trans-membrane adaptor	Strazza et al., 2021 (153)	Lu et al., 2017 (166) Dong et al., 2018 (167) Shen et al., 2018 (168)	Breast cancer, head/neck and cervical SCC, HCC, lung adenocarcinoma, uterine corpus endometrial carcinoma, CRC and melanoma (model)	0	N/A	N/A	N/A

Four of these proteins are already targeted in cancer clinical trials, two of which are also being studied in combination with immune checkpoint blockade.

^Papers establishing these findings.

*Trials on clinical trials.gov at the time of publication.

GI, gastrointestinal; NSCLC, non-small cell lung cancer; ALL, acute lymphocytic leukemia; HCC, hepatocellular carcinoma; CRC, colorectal carcinoma; SCC, squamous cell carcinoma; MDS, myelodysplastic syndrome; RAEB-T, Refractory Anemia With Excess Blasts in Transformation; TS, tumor suppressor. N/A is not available.

To address the currently limited response to checkpoint inhibitor therapy, combinational approaches already show great promise. With continued translational studies to further analyze PD-1 signaling, combinational strategies can improve response rates while mitigating adverse effects in cancer immunotherapies.

## Author Contributions

EM, MS, and AM designed, wrote, and edited the manuscript. All authors contributed to the article and approved the submitted version.

## Funding

This work was supported by grants from the NIH (AI125640, CA231277, AI150597).

## Conflict of Interest

The authors declare that the research was conducted in the absence of any commercial or financial relationships that could be construed as a potential conflict of interest.

## Publisher’s Note

All claims expressed in this article are solely those of the authors and do not necessarily represent those of their affiliated organizations, or those of the publisher, the editors and the reviewers. Any product that may be evaluated in this article, or claim that may be made by its manufacturer, is not guaranteed or endorsed by the publisher.
